# New insights gained from a decade long multi-center retrospective study on the management strategy for heterotopic pregnancy: expectant management is worth considering in patients without vaginal bleeding

**DOI:** 10.3389/fmed.2026.1766951

**Published:** 2026-05-13

**Authors:** Xiaoyue Chen, Yifeng Liu, Shufeng Kang, Wenpei Shi, Jing Ouyang, Tao Su, Shuangdi Li

**Affiliations:** 1 Shanghai Key Laboratory of Maternal Fetal Medicine, Department of Gynecology, Shanghai First Maternity and Infant Hospital, School of Medicine, Shanghai Institute of Maternal-Fetal Medicine and Gynecologic Oncology, Tongji University, Shanghai, China; 2 International Peace Maternity and Child Health Hospital, School of Medicine, Shanghai Jiao Tong University, Shanghai, China

**Keywords:** expectant management, heterotopic pregnancy, live birth, surgical intervention, vaginal bleeding

## Abstract

**Background:**

Heterotopic pregnancy (HP) is a rare but clinically challenging condition because treatment of the ectopic component must be balanced against preservation of the coexisting intrauterine pregnancy. The optimal management strategy remains controversial, particularly for clinically stable patients.

**Methods:**

This multicenter retrospective cohort study included women diagnosed with heterotopic pregnancy over a 10-year period. Patients were managed either expectantly or surgically according to clinical presentation, hemodynamic status, serial transvaginal ultrasound findings, and physician judgment. Baseline characteristics, treatment details, and intrauterine pregnancy outcomes were compared between groups. Multivariable logistic regression and subgroup interaction analyses were performed to explore factors associated with live birth.

**Results:**

A total of 159 women with heterotopic pregnancy were included, with a mean age of 31.9 ± 3.7 years. Overall, intrauterine pregnancy outcomes did not differ significantly between the operative and expectant management groups. However, patients selected for expectant management had more favorable baseline characteristics, including smaller ectopic masses, less pelvic fluid, higher hemoglobin levels, and absence of ectopic fetal cardiac activity. In subgroup analysis, among patients without vaginal bleeding, expectant management was associated with a higher live birth rate than surgical intervention (89.7% vs. 63.2%). Some subgroup estimates were accompanied by wide confidence intervals and should therefore be interpreted cautiously.

**Conclusion:**

Expectant management may be considered in carefully selected, hemodynamically stable patients with heterotopic pregnancy, particularly in those without vaginal bleeding. However, because of the retrospective design and baseline differences between treatment groups, these findings should be interpreted as supporting a feasible alternative in selected patients rather than proving superiority over surgical intervention.

## Introduction

Heterotopic pregnancy (HP), first described by Duverny in 1708, refers to the simultaneous occurrence of both an intrauterine and ectopic pregnancy (1). While rare in spontaneous pregnancies, with an incidence of approximately 1 in 30,000 (2), its prevalence rises to around 1% in cases involving ART (3).

Symptoms of HP are often non-specific, and abdominal pain and vaginal bleeding can be easily mistaken for other conditions related to intrauterine pregnancy (4). Transvaginal ultrasound serves as a valuable tool for early and accurate diagnosis (5). However, even with confirmation of an intrauterine pregnancy, the possibility of coexisting ectopic pregnancy remains (6). Due to challenges in early diagnosis, patients affected by HP face an elevated risk of complications such as tubal rupture, hypovolemic shock, and maternal or fetal death (7). Moreover, there is no universally accepted management strategy for HP. Various treatment options have been reported in published case series, including expectant management (8, 9), surgical intervention (10), and sonographically guided embryo aspiration (11) with or without embryo-killing drugs (12, 13). However, there is still no published high-quality clinical research. The ectopic gestational sac of HP can implant in different locations, such as the fallopian tube, uterine corner, uterine cervix, previous cesarean scar, or even in the abdomen (10), make the treatment complicated. Because many patients conceived through assisted reproductive technology, treatment selection needed to minimize adverse effects on the coexisting intrauterine pregnancy and future reproductive potential. In cases where intrauterine fetuses are viable, the treatment approach must carefully balance the safety of pregnant women and the wellbeing of the intrauterine pregnancy.

The objective of this retrospective study is to summarize the experiences of two tertiary maternity hospitals in Shanghai, China, regarding the management of HP and to analyze the impact of different treatment modalities on perinatal outcomes.

## Materials and methods

### Study design and setting

This was a retrospective, multicenter cohort study conducted at two tertiary maternity hospitals in Shanghai, China: the Obstetrics & Gynecology Hospital of Tongji University and the International Peace Maternity and Child Health Hospital affiliated with Shanghai Jiao Tong University. The study period extended from May 2014 to October 2023.

Ethical approval was obtained from the ethics committees of the participating hospitals. The study protocol was reviewed and approved by the Ethics Committee of Shanghai First Maternity and Infant Hospital (Approval No. KS23139), which served as the coordinating review board for all participating sites. Because this was a retrospective study based primarily on de-identified clinical data, the requirement for written informed consent for chart review was waived by the ethics committee. The ethics-approved protocol also authorized the research team, when necessary, to contact patients to supplement missing obstetric and neonatal outcome data. When telephone follow-up was performed, verbal informed consent was obtained from the contacted women before additional information was collected.

### Study population and data collection

Women diagnosed with HP during the study period were identified through institutional medical records. Heterotopic pregnancy was defined as the simultaneous presence of an intrauterine gestational sac and an extrauterine gestational sac or ectopic mass, confirmed by transvaginal ultrasound and/or surgical findings. Diagnosis was based on transvaginal ultrasound findings, intraoperative confirmation, and/or histological examination of ectopic tissue when available.

Eligible patients were classified into two groups according to the initial management strategy: expectant management and operative intervention. The two participating centers followed broadly similar diagnostic and management principles for heterotopic pregnancy. To minimize inter-center heterogeneity, all cases were reviewed using a unified data extraction form and common outcome definitions.

Clinical charts were reviewed in detail. Data collected included maternal age, parity, conception method, gestational age at diagnosis, presenting symptoms, ultrasound findings, laboratory test results, treatment strategy, and intrauterine pregnancy outcomes. Obstetric and neonatal outcomes were confirmed through medical records and supplemented, when necessary, by telephone follow-up through October 2023. All medical records and sonographic images were reviewed to minimize misclassification.

### Ultrasound assessment

All patients underwent transvaginal ultrasound at diagnosis. Ultrasound assessment included the location and maximal diameter of the ectopic mass, the presence or absence of a yolk sac and fetal cardiac activity in the ectopic component, the amount of pelvic fluid, and the status of the intrauterine pregnancy. All transvaginal ultrasound examinations were performed and interpreted by experienced sonographers or gynecologists at the participating tertiary centers.

An ectopic mass was diagnosed based on the presence of an adnexal gestational sac-like structure, a yolk sac or embryo with or without cardiac activity, or a heterogeneous adnexal mass separate from the ovary, together with serial imaging findings and clinical correlation, in order to differentiate it from a corpus luteum cyst.

### Treatment strategy

Management strategies included expectant management or operative intervention. Treatment decisions were made according to symptoms, hemodynamic stability, serial transvaginal ultrasound findings, and the clinical judgment of the treating physician.

Expectant management was considered for clinically stable patients without evidence of active intra-abdominal bleeding, severe abdominal pain, or clear signs of rupture, particularly when the ectopic component showed no fetal cardiac activity and close follow-up could be ensured. In this study, expectant management was defined as close clinical and ultrasound follow-up without active medical or surgical intervention. Medical management, including local potassium chloride injection or other embryo reduction procedures, was not categorized as expectant management.

Failure of expectant management was defined as the occurrence of worsening abdominal pain, increasing pelvic fluid, progressive enlargement of the ectopic mass on serial ultrasound, declining hemoglobin levels, hemodynamic instability, or patient preference for intervention. Patients who initially underwent expectant management but subsequently required surgical intervention were recorded as crossover cases.

Operative intervention was performed when there was concern for rupture, significant symptoms, hemodynamic instability, progressive enlargement of the ectopic mass, or ongoing intra-abdominal bleeding. Surgical indications also included progressive enlargement of the ectopic mass to more than 3 cm and/or internal bleeding or declining hemoglobin levels. Laparoscopic surgical intervention was performed under intravenous anesthesia with CO2 pneumoperitoneum pressure maintained below 12 mmHg. Postoperatively, oral dydrogesterone was routinely administered, whereas prophylactic antibiotics were not routinely used.

### Outcome measures

The primary outcome was live birth of the intrauterine pregnancy. Secondary outcomes included miscarriage, ongoing pregnancy, neonatal birthweight, neonatal length, Apgar scores, and treatment crossover from expectant management to surgical intervention.

### Statistical analysis

Continuous variables were expressed as mean ± standard deviation or median (interquartile range), as appropriate, and categorical variables were presented as counts and percentages. Continuous variables were compared using Student’s *t*-test or the Mann–Whitney U test, and categorical variables were compared using the chi-square test or Fisher’s exact test, as appropriate. Univariable and multivariable logistic regression analyses were performed to explore factors associated with intrauterine pregnancy outcomes. Interaction analyses were further conducted to assess whether the association between treatment strategy and intrauterine pregnancy outcome differed across clinically relevant subgroups, including vaginal bleeding status. Because some subgroup strata contained relatively small numbers of patients, the corresponding odds ratios and confidence intervals may be unstable and were therefore interpreted cautiously. A two-sided *P* < 0.05 was considered statistically significant. All statistical analyses were performed using R version 4.0.3.

## Results

### Baseline characteristics of study participants

This study included 159 patients from two hospitals with an average age of 31.9 ± 3.7 years, with 79.9% being < 35 years old. 88.7% were primiparous, and 80.5% had no previous history of ectopic pregnancy. Among the HP patients, 26 had natural pregnancies, while 133 cases resulted from assisted reproductive technology (ART). Patients presented with lower abdominal pain in 40.3% of cases, while vaginal bleeding occurred in 62.3% of cases. Ultrasound examinations revealed that 76.1% of ectopic pregnancies located at the adnexa, with the majority lacking a yolk sac (95%) or fetal heart activity (97.5%). Table 1 presents the clinical characteristics of HP patients.

**TABLE 1:** General maternal characteristics, symptoms, and diagnostic features.

Characteristic	Number of patients
	(*n* = 159)
Age (years)	
Mean (SD)	31.9 (3.70)
Median [min, max]	32.0 [23.0, 44.0]
< 35	127 (79.9%)
≥ 35	32 (20.1%)
Gravidity	
1	90 (56.6%)
2	31 (19.5%)
3	38 (23.9%)
Parity	
Primiparity	141 (88.7%)
Multiparity	18 (11.3%)
History of ectopic pregnancy (times)	
0	128 (80.5%)
1	24 (15.1%)
2	6 (3.8%)
3	1 (0.6%)
Previous C-section history	
Yes	8 (5.0%)
No	151 (95.0%)
Method of fertilization	
Natural conception	26 (16.4%)
Assisted reproductive technology(ART)	133 (83.6%)
Ovulation induction	12 (7.5%)
IVF-ET	121 (76.1%)
Number of transferred embryos	
Mean (SD)	1.99 (0.0909)
Median [Min, Max]	2.00 [1.00, 2.00]
N/A	38 (23.9%)
Frozen–thawed embryo transferred	
Yes	102 (64.2%)
No	19 (11.9%)
N/A	38 (23.9%)
Gestational age at diagnosis (days)	
Mean (SD)	51.4 (15.4)
Median [Min, Max]	49.0 [30.0, 141]
Lower abdominal pain	
Yes	64 (40.3%)
No	95 (59.7%)
Vaginal bleeding	
Yes	99 (62.3%)
No	60 (37.7%)
Ultrasound findings	
Location of ectopic pregnancy	
Left Adnexal	58 (36.5%)
Right Adnexal	63 (39.6%)
Cervix or uterine horns	16 (10.1%)
N/A	22 (13.8%)
Yolk sac	
No	151 (95.0%)
Yes	8 (5.0%)
Fetal cardiac activity	
No	155 (97.5%)
Yes	4 (2.5%)
Maximum diameter of the ectopic mass (mm)	
Mean (SD)	30.4 (17.8)
Median [Min, Max]	24.0 [6.00, 96.0]
Maximum depth of pelvic fluid (mm)	
Mean (SD)	17.1 (15.5)
Median [Min, Max]	16.0 [0, 71.0]
Laboratory test	
Hemoglobin	
Mean (SD)	124 (11.1)
Median [Min, Max]	125 [90.0, 151]
Missing	7 (4.4%)
Platelet count (× 10^9^/L)	
Mean (SD)	246 (59.3)
Median [Min, Max]	244 [118, 475]
Missing	7 (4.4%)
HCG growth rate per day	
Mean (SD)	2.65 (4.73)
Median [Min, Max]	1.28 [0.00236, 32.8]
Missing	36 (22.6%)
D-dimer	
Mean (SD)	0.598 (0.814)
Median [Min, Max]	0.320 [0.100, 6.95]

N/A indicates cases in which the relevant information could not be reliably determined from the available clinical records or imaging documentation. For the location of ectopic pregnancy, N/A indicates that the exact ectopic implantation site could not be reliably identified from the available medical records and imaging data.

### Comparison between expectant management and surgical intervention

Out of 159 HP patients, 77 received surgical treatment, while 82 underwent expectant management. Patients presenting with lower abdominal pain (OR = 2.32; 95%CI [1.22;4.50]; *p* = 0.015), larger diameter of the ectopic mass (OR = 1.07; 95%CI [1.04;1.10]; *p* < 0.001), more pelvic fluid (OR = 1.06; 95% CI [1.03;1.08]; *p* < 0.001), lower hemoglobin levels (OR = 0.96; 95%CI[0.93;0.99]; *p* = 0.004), and higher neutrophil counts (OR = 1.23; 95%CI [1.08;1.39]; *p* < 0.001) were more likely to undergo surgical intervention. However, there were no significant differences observed in intrauterine pregnancy outcomes, birth weight, neonatal length, or Apgar scores, as shown in Table 2.

**TABLE 2:** Comparisons between expectant management and surgical intervention.

Characteristic	Expectant management (*n* = 82)	Surgical intervention (*n* = 77)	OR (95% CI)	*P-*value
Age Median [min, max]	31.0 [29.0;34.0]	32.0 [30.0;34.0]	0.97 [0.64;1.48]	0.811
≥ 35				0.693
No	64 (78.0%)	63 (81.8%)	Ref.	
Yes	18 (22.0%)	14 (18.2%)	0.79 [0.36;1.74]	
Gravidity				0.006*
1	56 (68.3%)	34 (44.2%)	Ref.	
2	10 (12.2%)	21 (27.3%)	3.40 [1.45;8.44]	
3	16 (19.5%)	22 (28.6%)	2.24 [1.04;4.95]	
Parity				0.058
Primiparity	5 (6.10%)	13 (16.9%)	Ref.	
Multiparity	77 (93.9%)	64 (83.1%)	0.33 [0.10;0.93]	
Method of fertilization				0.640
Natural conception	15 (18.3%)	11 (14.3%)	Ref.	
ART	67 (81.7%)	66 (85.7%)	1.34 [0.57;3.22]	
History of ectopic pregnancy				0.162
No	70 (85.4%)	58 (75.3%)	Ref.	
Yes	12 (14.6%)	19 (24.7%)	1.90 [0.85;4.36]	
Previous C-section history:				0.485
No	79 (96.3%)	72 (93.5%)	Ref.	
Yes	3 (3.66%)	5 (6.49%)	1.79 [0.41;9.55]	
Lower abdominal pain				0.015*
No	57 (69.5%)	38 (49.4%)	Ref.	
Yes	25 (30.5%)	39 (50.6%)	2.32 [1.22;4.50]	
Vaginal bleeding:				0.069
No	37 (45.1%)	23 (29.9%)	Ref.	
Yes	45 (54.9%)	54 (70.1%)	1.92 [1.00;3.74]	
Location of ectopic pregnancy				0.082
Bilateral Adnexal	70 (93.3%)	51 (82.3%)	Ref.	
Others	5 (6.67%)	11 (17.7%)	2.95 [0.99;10.1]	
Yolk sac				0.002
No	82 (100%)	69 (89.6%)	Ref.	
Yes	0 (0.00%)	8 (10.4%)		
Fetal Cardiac activity				0.053
No	82 (100%)	73 (94.8%)	Ref.	
Yes	0 (0.00%)	4 (5.19%)		
The maximum diameter of the ectopic mass	23.4 (10.5)	37.8 (20.7)	1.07 [1.04;1.10]	< 0.001
The maximum depth of pelvic fluid	11.7 (11.1)	22.9 (17.3)	1.06 [1.03;1.08]	< 0.001
Hemoglobin	127 (9.38)	122 (12.2)	0.96 [0.93;0.99]	0.004
Platelet	242 (57.5)	251 (61.2)	1.00 [1.00;1.01]	0.327
Number of neutrophils	6.55 (2.10)	8.27 (3.81)	1.23 [1.08;1.39]	0.001
hcg2/hcg1/(time2-time1)	2.49 (4.07)	2.82 (5.39)	1.01 [0.94;1.09]	0.708
Intrauterine pregnancy outcome				0.577
Miscarriage	14 (20.9%)	17 (26.6%)	Ref.	
Live birth	53 (79.1%)	47 (73.4%)	0.73 [0.32;1.66]	
Birth weight	3170 (582)	3198 (544)	1.00 [1.00;1.00]	0.829
Neonatal length	49.4 (2.38)	49.5 (1.92)	1.02 [0.82;1.28]	0.837
APGAR score				0.354
10–10	25 (69.4%)	21 (67.7%)	Ref.	
8–10	3 (8.33%)	0 (0.00%)		
8–9	1 (2.78%)	0 (0.00%)		
9–10	5 (13.9%)	8 (25.8%)		
9–9	2 (5.56%)	2 (6.45%)		

Baseline characteristics were compared in the overall cohort of 159 patients. Intrauterine pregnancy outcome analyses in this table were based on 131 patients with available outcome data.

Among patients initially managed expectantly, 10 subsequently required surgical intervention during follow-up because of worsening clinical symptoms, sonographic progression of the ectopic component, and/or patient preference for intervention. In the expectant management group, intrauterine pregnancy losses were not associated with progression or rupture of the heterotopic mass. No intrauterine pregnancy loss was directly attributable to rupture or progression of the ectopic component, and all were considered independent spontaneous miscarriages.

### Pregnancy outcomes

Among the 159 patients included in the overall cohort, 131 had available intrauterine pregnancy outcome data and were included in the outcome analyses, whereas 28 were excluded because of incomplete clinical records or inability to confirm pregnancy outcomes through telephone follow-up. These 28 patients included 17 cases from Shanghai First Maternity and Infant Hospital and 11 cases from the International Peace Maternity and Child Health Hospital.

**FIGURE 1:**
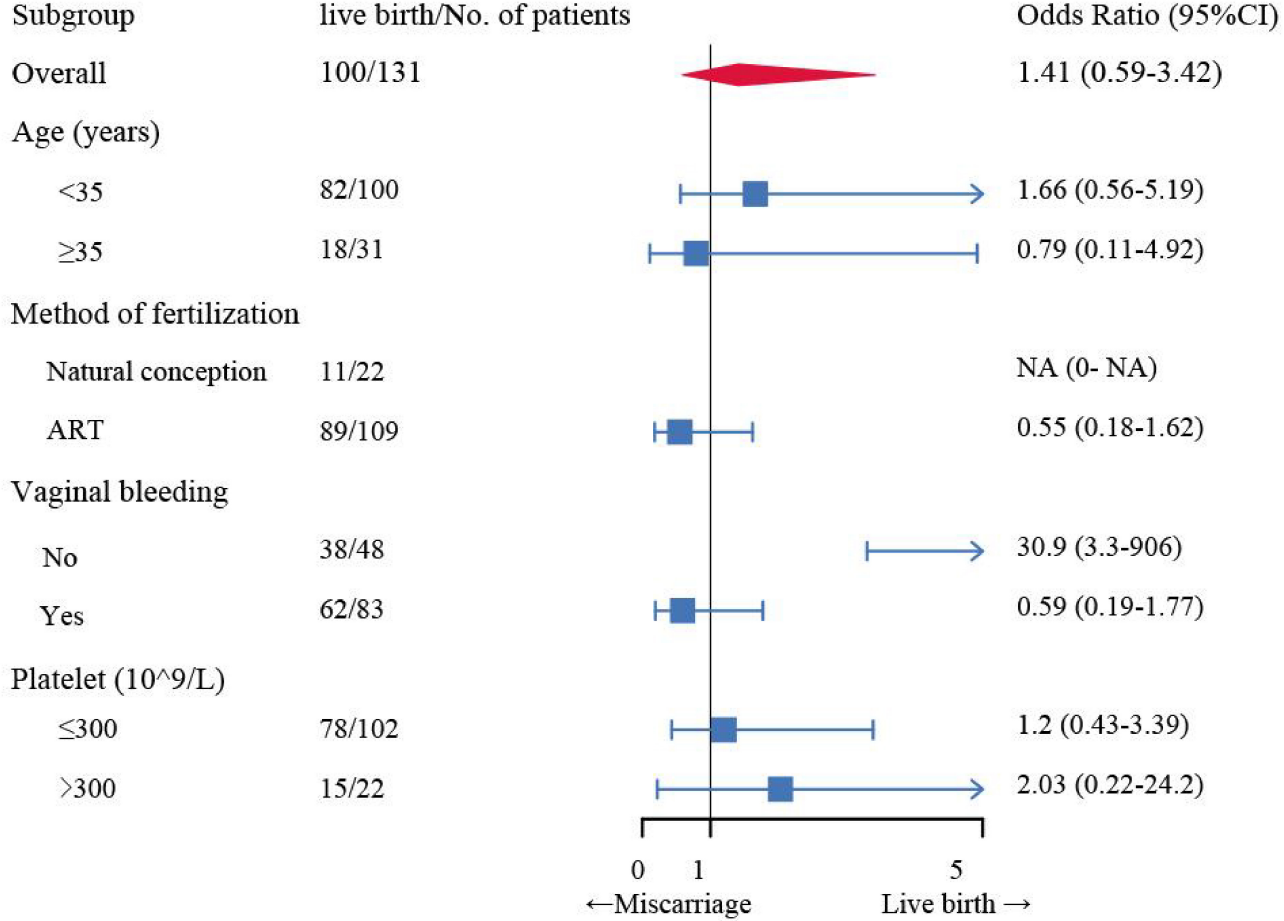
Subgroup analysis of the association between treatment strategy and intrauterine pregnancy outcome. Among patients without vaginal bleeding, expectant management was associated with a higher live birth rate than surgical intervention. ART, Assisted reproductive technology; CI, confidence interval. The adjusted odds ratios and 95% confidence intervals were calculated by adjusting treatment, age, method of fertilization, vaginal bleeding, and platelet.

Among the 131 patients included in the outcome analyses, 31 experienced miscarriage and 100 had a live birth. Those who had a live birth were younger (*p* = 0.001), more likely to be multiparous (*p* = 0.028), underwent pregnancy with assisted reproductive technology (*p* = 0.004), and had higher serum hCG levels (*p* = 0.037) and progesterone levels (*p* = 0.002), as shown in Table 3. We further constructed multivariate logistic analysis models to study the factors related to intrauterine pregnancy outcomes, as shown in Table 4. Additionally, subgroup and interaction analyses were performed to stratify the relevance between intrauterine pregnancy outcomes and related factors (Table 5), as illustrated in Figure 1.

**TABLE 3:** Comparison between miscarriage and live birth in 131 patients.

Characteristic	Miscarriage (*n* = 31)	Live birth (*n* = 100)	OR (95% CI)	*P-*value
Age	34.0 (3.30)	31.6 (3.69)	0.40 [0.22;0.73]	0.001
≥ 35				0.012
No	18 (58.1%)	82 (82.0%)	Ref.	
Yes	13 (41.9%)	18 (18.0%)	0.31 [0.13;0.75]	
Gravidity				0.723
1	17 (54.8%)	60 (60.0%)	Ref.	
2	5 (16.1%)	18 (18.0%)	1.00 [0.34;3.47]	
3	9 (29.0%)	22 (22.0%)	0.69 [0.27;1.85]	
Parity				0.028
Primiparity	8 (25.8%)	9 (9.00%)	Ref.	
Multiparity	23 (74.2%)	91 (91.0%)	3.48 [1.17;10.3]	
ART				0.004 0.459
No	11 (35.5%)	11 (11.0%)	Ref.	
Yes	20 (64.5%)	89 (89.0%)	4.38 [1.64;11.8]	
History of ectopic pregnancy				
No	27 (87.1%)	79 (79.0%)	Ref.	
Yes	4 (12.9%)	21 (21.0%)	1.74 [0.59;6.56]	
Lower abdominal pain				0.907
No	19 (61.3%)	58 (58.0%)	Ref.	
Yes	12 (38.7%)	42 (42.0%)	1.14 [0.50;2.68]	
Vaginal bleeding:				0.714
No	10 (32.3%)	38 (38.0%)	Ref.	
Yes	21 (67.7%)	62 (62.0%)	0.78 [0.32;1.82]	
Location of ectopic pregnancy				0.014
Others	3 (10.7%)	9 (10.6%)	Ref.	
Right adnexal	19 (67.9%)	33 (38.8%)	0.60 [0.12;2.36]	
Left adnexal	6 (21.4%)	43 (50.6%)	2.39 [0.41;11.4]	
Yolk sac				0.669
No	29 (93.5%)	95 (95.0%)	Ref.	
Yes	2 (6.45%)	5 (5.00%)	0.73 [0.14;5.95]	
Fetal Cardiac activity				0.558
No	30 (96.8%)	98 (98.0%)	Ref.	
Yes	1 (3.23%)	2 (2.00%)	0.58 [0.05;18.6]	
The maximum diameter of the ectopic mass	27.3 (17.2)	31.7 (18.8)	1.01 [0.99;1.04]	0.227
The maximum depth of pelvic fluid	20.8 (16.0)	16.0 (15.2)	0.98 [0.96;1.01]	0.148
Hemoglobin	123 (9.77)	125 (11.6)	1.01 [0.98;1.05]	0.439
Platelet	261 (75.2)	239 (52.8)	0.99 [0.99;1.00]	0.139
Hcg	18,239 (32869)	37,028 (63506)	1.00 [1.00;1.00]	0.037
P	62.9 (47.5)	131 (109)	1.02 [1.00;1.03]	0.002
hcg2/hcg1/(time2-time1)	1.91 (2.27)	2.90 (5.33)	1.07 [0.92;1.26]	0.192

**TABLE 4:** Multivariable logistic regression for intrauterine pregnancy outcome in 131 patients.

	Model A		Model B		Model C		Model D	
Surgical intervention	Ref							
Expectant	1.38 (0.41, 4.57)		1.55(0.37, 6.60)		1.52 (0.34, 6.94)		0.44 (0.04, 3.69)	
Characteristic	OR (95%CI)	*p*-value	OR (95%CI)	*p*-value	OR (95% CI)	*p*-value	OR (95% CI)	*p*-value
Treatment		0.806		0.393		0.493		0.562
surgical intervention								
Expectant	1.16(0.36, 3.67)		1.77(0.47, 6.82)		1.62(0.41, 6.59)		0.58(0.08, 3.56)	
Age ≥ 35		0.012		0.004		0.006		0.005
No								
Yes	0.22 (0.07, 0.72)		0.15(0.04, 0.56)		0.16(0.04, 0.59)		0.11(0.02, 0.51)	
Parity		0.447		0.555		0.642		0.303
Primiparity								
Multiparity	1.85(0.36, 8.97)		1.64(0.30, 8.27)		1.54(0.23, 9.15)		3.2(0.33, 32.3)	
ART		0.008		0.012		0.005		0.001
No								
Yes	7.9(1.72, 46.1)		7.64(1.56, 48.0)		11.5(2.01, 88.3)		30(3.62, 501)	
Location of ectopic pregnancy		0.679		0.728		0.527		0.816
Bilateral Adnexal								
Others	1.85(0.13, 66.4)		1.66(0.12, 59.0)		2.53(0.17, 89.2)		0.63(0.01, 41.4)	
Vaginal bleeding:		0.037		0.034		0.02		0.067
No								
Yes	0.23(0.04, 0.9	0.314	0.22(0.04, 0.90)	0.122	0.18(0.03, 0.78)	0.041	0.22(0.03, 1.11)	0.3
The maximum diameter of the ectopic mass	1.06 (0.95, 1.28)		1.04(0.99, 1.09)		1.06(1.00, 1.12)		1.04(0.97, 1.12)	
The maximum depth of pelvic fluid			1(0.96, 1.05)	0.903	1(0.95, 1.05)	0.992	1.04(0.97, 1.12)	0.27
hcg_p_d			1.06(0.96, 1.28)	0.281	1.08(0.97, 1.30)	0.177	1.14(0.99, 1.65)	0.066
Hemoglobin					1.04(0.98, 1.10)	0.171	1.04(0.98, 1.13)	0.212
Neutrophil					0.93(0.76, 1.14)	0.446	0.97(0.75, 1.24)	0.775
Platelet							0.98(0.96, 1.00)	0.006
Lymphocytes							1.07(0.22, 5.35)	0.93
D-dimer							0.32(0.02, 4.65)	0.386

OR, odds ratio; CI, confidence interval.

**TABLE 5:** Interaction analysis of vaginal bleeding and treatment strategy among 131 patients.

Characteristic	OR^1^	95% CI^1^	*p*-value
Treatment			0.004
Surgical intervention			
Expectant	11	2.06, 76.2	0.046
Age ≥ 35	0.36	0.13, 0.98	
No			
Yes			
ART	7.12	2.21, 25.7	< 0.001
No			0.122
Yes			
Vaginal bleeding:	2.97	0.75, 12.1	
No			0.032
Yes			
Platelet	0.99	0.98, 1.00	
Interaction analysis of vaginal bleeding and treatment	0.05	0.00, 0.36	0.003
Expectant management			

^1^OR, odds ratio; CI, confidence interval.

The impact of treatment option on intrauterine pregnancy outcomes showed no difference in subgroups with different clinical characteristics such as age, conception mode and platelets. Expectant management was found to be acceptable in heterotopic pregnancy, with a higher live birth rate observed in those patients without vaginal bleeding compared to those who underwent surgical intervention. Subgroup analyses revealed that among patients without vaginal bleeding, expectant management was associated with a higher live birth rate (89.7% vs. 63.2%).

**FIGURE 2:**
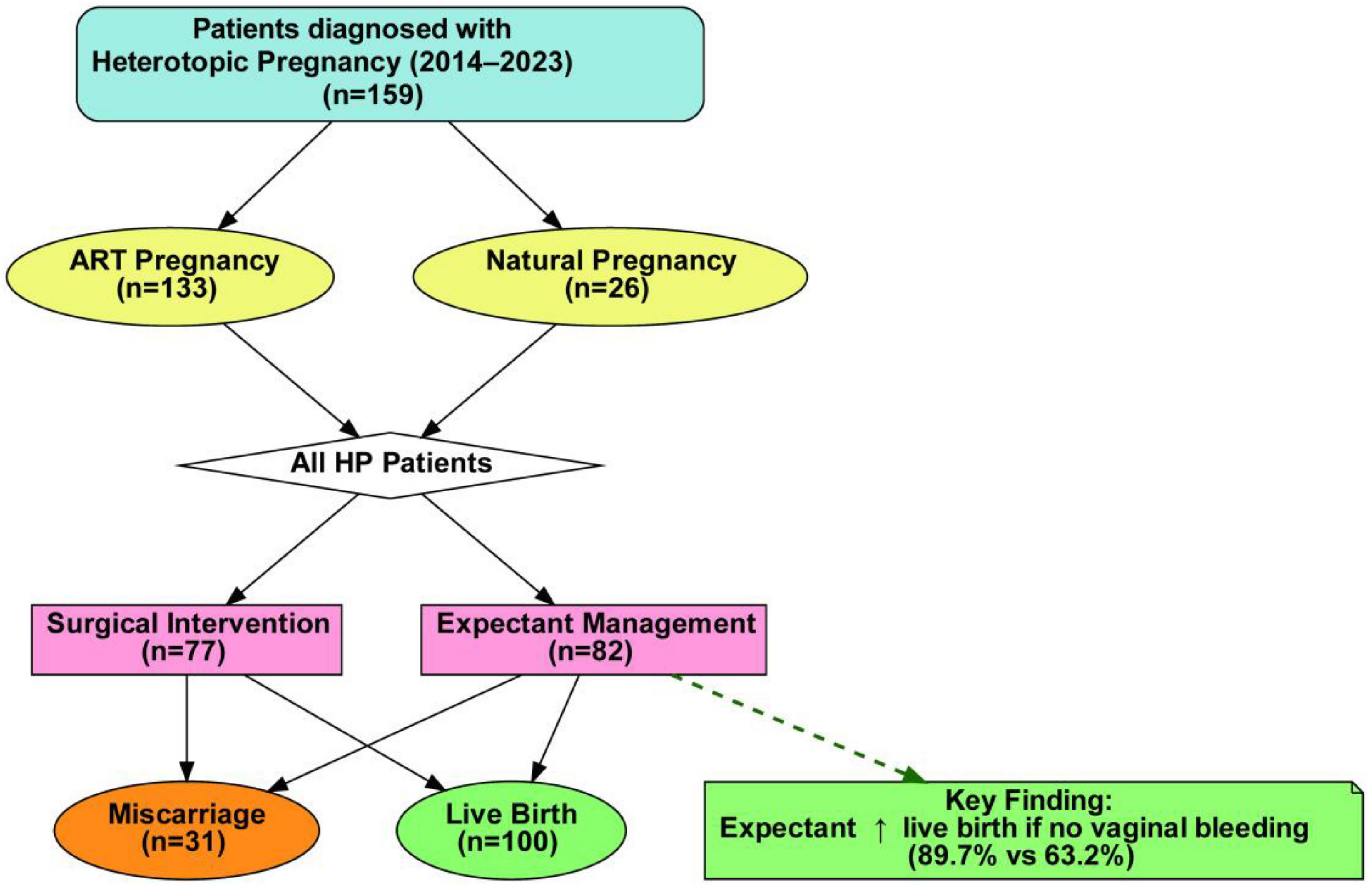
Flowchart of patient inclusion, management strategies, and intrauterine pregnancy outcomes in heterotopic pregnancy. Among 159 patients, 77 underwent surgical intervention and 82 received expectant management. A total of 131 patients had available intrauterine pregnancy outcome data and were included in the outcome analyses, whereas 28 were excluded because of incomplete clinical records or inability to confirm outcomes through telephone follow-up. *P < 0.05.

Figure 2 shows a flowchart summarizing the management strategies (surgical vs. expectant) and intrauterine pregnancy outcomes (miscarriage vs. live birth). Importantly, it highlights the key finding that in patients without vaginal bleeding, expectant management was associated with a significantly higher live birth rate.

## Discussion

Heterotopic pregnancy (HP), although rare, remains a clinically challenging condition in both diagnosis and management. Most of the available literature consists of case reports or small case series, which limits the generalizability of published findings (14–18). In this context, our multicenter retrospective cohort, spanning nearly 10 years and including 159 patients, represents one of the larger reported series on HP. The main clinical question addressed in this study was whether different management strategies influence the outcome of the coexisting intrauterine pregnancy.

Early diagnosis of HP remains difficult because the presenting symptoms are often non-specific. Abdominal pain and vaginal bleeding are the most common complaints and can easily be mistaken for symptoms related to threatened miscarriage or isolated ectopic pregnancy (19, 20). In our cohort, 62.3% of patients had vaginal bleeding and 40.3% reported lower abdominal pain. These proportions differ somewhat from previous reports, which may be related to the fact that both participating centers are specialized tertiary maternity hospitals with advanced ultrasound expertise and that a large proportion of our patients conceived through assisted reproductive technology (ART), resulting in earlier and more frequent ultrasound evaluation. The increasing availability of high-quality transvaginal ultrasound has substantially improved the diagnostic accuracy of HP; however, confirmation of an intrauterine pregnancy does not exclude the coexistence of an ectopic pregnancy (21–23). Therefore, a high index of suspicion, careful clinical assessment, and routine early ultrasound remain essential for timely diagnosis.

Once diagnosed, management of HP requires balancing maternal safety against preservation of the intrauterine pregnancy. In current clinical practice, available strategies include expectant management, operative intervention, and, in selected settings, ultrasound-guided embryo reduction or local injection therapy (12, 24–29). In our study, the two most commonly used approaches were surgical intervention and expectant management, whereas ultrasound-guided selective embryo reduction was rarely performed. This likely reflects local practice patterns, technical availability, and differences in treatment routines. Our findings suggest that, at present, surgical intervention and expectant management remain the principal management strategies for HP in our clinical setting.

In the overall cohort, no significant difference was observed between expectant management and operative intervention with respect to intrauterine pregnancy outcomes. However, in subgroup analysis, expectant management was associated with a higher live birth rate among patients without vaginal bleeding. This finding is clinically interesting because vaginal bleeding is a simple and readily available bedside feature that may help identify a subset of patients in whom conservative management could be considered.

The lower live birth rate observed in the surgical group may also have several explanations. First, operative intervention is usually reserved for patients with more severe symptoms, greater clinical concern, or less favorable baseline conditions, all of which may themselves adversely affect intrauterine pregnancy outcomes. Second, anesthesia exposure, surgical stress, manipulation of the uterus and adnexa, and postoperative inflammatory responses may theoretically contribute to pregnancy loss (30), although these mechanisms could not be directly assessed in the present study. Therefore, the poorer pregnancy outcomes observed in the operative group should not be attributed solely to the surgical procedure itself.

Another important issue is the definition and safety profile of expectant management. In clinical practice, expectant management should only be considered in hemodynamically stable patients in whom serial ultrasound and close clinical monitoring can be ensured. Its safety depends not only on favorable baseline characteristics but also on timely recognition of worsening symptoms or sonographic progression (24). In our study, some patients initially managed expectantly subsequently required surgical intervention, highlighting the importance of strict follow-up and clear criteria for treatment conversion. Accordingly, expectant management should not be regarded as passive expectant management, but rather as an active surveillance strategy in carefully selected patients.

In our cohort, most ectopic components lacked a yolk sac or fetal cardiac activity. This may reflect early detection, particularly in a population with a high proportion of ART pregnancies undergoing close sonographic surveillance at tertiary referral centers, rather than a universal sonographic feature of heterotopic pregnancy (17, 31). Published data specifically quantifying the prevalence of these ectopic intrasac structures in heterotopic pregnancy remain limited, and this finding should therefore be interpreted in the context of our study population.

This study has several limitations. First, its retrospective design introduces selection bias and confounding by indication. Patients managed expectantly were generally more stable at baseline, with smaller ectopic masses, less pelvic fluid, higher hemoglobin levels, and no ectopic fetal cardiac activity. Therefore, the more favorable outcomes observed in this group may partly reflect selection of better candidates for conservative treatment rather than a true superiority of expectant management over surgical intervention. Accordingly, the subgroup finding of a higher live birth rate among patients without vaginal bleeding should also be interpreted cautiously. Second, although this study represents one of the larger reported cohorts of heterotopic pregnancy, the sample size remained limited for some subgroup analyses, leading to wide confidence intervals and reduced precision of effect estimates. Third, the multicenter design may have introduced some heterogeneity in management decisions. In addition, because delivery occurred at different hospitals in some cases, detailed gestational age at delivery was not consistently available in the database, which limited our ability to compare preterm delivery rates and gestational week at delivery between groups. Finally, because treatment allocation was not randomized, causal inference cannot be established.

Despite these limitations, the present study provides clinically useful information for a rare and difficult obstetric scenario. Our results suggest that the presence or absence of vaginal bleeding may help refine risk stratification and clinical decision-making, and that expectant management may be a reasonable alternative in selected hemodynamically stable patients with heterotopic pregnancy. Future prospective studies with standardized management criteria are needed to confirm which patients are most likely to benefit from expectant management and to further optimize individualized treatment strategies.

## Conclusion

In conclusion, this multicenter retrospective study suggests that expectant management may be a feasible option in carefully selected, hemodynamically stable patients with heterotopic pregnancy, particularly in those without vaginal bleeding. However, because patients managed expectantly had more favorable baseline characteristics, these findings should be interpreted cautiously and should not be taken as evidence of superiority over surgical intervention. Rather, they support expectant management as a potential alternative in selected patients under close clinical and ultrasound surveillance. Further prospective studies are needed to establish standardized criteria for treatment selection and to identify which patients are most likely to benefit from expectant management.

## Data Availability

The raw data supporting the conclusions of this article will be made available by the authors, without undue reservation.
